# On the black slope: analysis of the course of a blunt renal trauma collective in a winter sports region

**DOI:** 10.1007/s00068-021-01830-w

**Published:** 2021-12-16

**Authors:** Christian Deininger, Thomas Freude, Florian Wichlas, Lukas Konstantin Kriechbaumer, Sebastian Hubertus Markus Deininger, Peter Törzsök, Lukas Lusuardi, Maximilian Pallauf, Amelie Deluca, Susanne Deininger

**Affiliations:** 1 Department of Orthopedics and Traumatology, Salzburg University Hospital, Paracelsus Medical University, Salzburg, Austria; 2 Department of Urology and Andrology, Salzburg University Hospital, Paracelsus Medical University, Salzburg, Austria; 3 Institute of Tendon and Bone Regeneration, Spinal Cord Injury and Tissue Regeneration Center Salzburg, Paracelsus Medical University, Salzburg, Austria

**Keywords:** Renal trauma, Winter sports, Abdominal, Skiing, Conservative, Non-operative management

## Abstract

**Purpose:**

The aim of this study was to analyze the injury patterns and clinical course of a winter sport dominated by blunt renal trauma collective.

**Methods:**

Blunt renal trauma cases (*N* = 106) treated in a Level 1 Trauma Center in Austria were analyzed.

**Results:**

We encountered 12.3% grade 1, 10.4% grade 2, 32.1% grade 3, 38.7% grade 4 and 6.6% grade 5 renal traumata classified according to the *American Association for the Surgery of Trauma* (AAST). The mechanisms of injury (MOI) did not have an influence on the frequency of HG trauma (i.e., grade 4 and 5). No concomitant injuries (CIs) were found in 57.9% of patients. The number of patients without CIs was significantly higher in the sports associated trauma group compared to other MOIs (*p* < 0.01). In 94.3% the primary treatment was a non-operative management (NOM) including 56.6% conservative, 34.0% endourological, and 3.8% interventional therapies. A follow-up computed tomography (FU-CT) was performed in 81.1%, 3.3 days after trauma. After FU-CT, the primary therapy was changed in 11.4% of cases (grade ≥ 3). Comparing the Hb loss between the patients with grade 3 and 4 kidney trauma with and without revision surgery, we find a significantly increased Hb loss within the first 96 h after the trauma in the group with a needed change of therapy (*p* < 0.0001). The overall rate of nephrectomy (primary or secondary) was 9.4%. Independent predictors of nephrectomy were HG trauma (*p* < 0.01), age (*p* < 0.05), and sex (*p* < 0.05). The probability of nephrectomy was lower with (winter) sports-associated trauma (*p* < 0.1).

**Conclusions:**

Sports-associated blunt renal trauma is more likely to occur isolated, and has a lower risk of severe outcomes, compared to other trauma mechanisms. NOM can successfully be performed in over 90% of all trauma grades.

**Supplementary Information:**

The online version contains supplementary material available at 10.1007/s00068-021-01830-w.

## Introduction

With up to 13% of all abdominal trauma cases, renal laceration is one of the most common intra-abdominal injuries in Western countries [1]. Renal traumata can occur isolated or in combination with other thoraco-abdominal organ injuries (spleen and liver) or rib fractures [2]. In up to 90% of cases, the mechanism of injury (MOI) is a blunt trauma [3], whereas penetrating traumata, such as stab wounds or gunshots, are rarely encountered in the Western World. Patients with renal lacerations are predominantly younger, with a mean age of 30.8 years, and 72% are male [4]. There are various blunt trauma mechanisms that have been connected to renal trauma: motor vehicle accidents, low falls, sport activities, and pedestrian accidents.

The location of the university hospital is in a leisure and winter sports region, which serves as a weekend and vacation destination for national and international guests. As a level 1 trauma center (TC), the hospital not only treats trauma patients from the city it is located in, but also severely injured from surrounding smaller hospitals (level II and III TCs). Thus, the amount of renal traumata requiring medical care and further evaluation is high, especially during the skiing season.

Among these cases, many receive a kidney preserving, i.e., Non Operative Management (NOM), including completely conservative, endourological (placement of Mono- or Double-J stent or nephrostomy tube) or interventional (coil-embolization) therapies.

The key question of this manuscript is how the injury patterns and clinical courses of trauma patients after sports and winter sports accidents turn out compared to trauma patients after different MOIs. In addition, the question arises, which patients received a Follow Up-Computed Tomography (FU-CT) during the clinical course and to what extent the CT influenced the therapy decision. The European Association of Urology (EAU) guideline recommends a FU-CT 2–4 days after high-grade (HG) or penetrating renal trauma, as well as in case of clinical deterioration [5]. Nephrectomy is certainly the treatment with the most far-reaching consequences. In this context, the identification of patients at high risk of nephrectomy is of vital importance. In addition, the young patient population here benefits from omitting unnecessary imaging.

The aim of this study was to analyze the clinical course of a winter sports dominated trauma collective, in particular the importance of FU-CT imaging and how it can predict the necessity for a delayed nephrectomy.

## Materials and methods

### Materials

The study was conducted as a collaboration between the Clinic for Urology and Andrology and the Clinic for Orthopedics and Traumatology of a Level 1 TC. A retrospective data analysis of blunt renal trauma patients between January 2010 and March 2020 was conducted. The consent of the local ethics commission was obtained. The approval number is 1078/2020. Patients’ basic demographics are shown in Table 1.

**Table 1:** Patients’ basic demographics

Variable	Mean	Median	SD	Min	Max
Age	43.0	44	20.0	15	88
Sex (share of male patients in percent)^a^	83.0				
Primary treatment (percent)^a^					
Open surgical/nephrectomy	5.7				
Endourological	33.9				
Conservative	56.6				
Interventional	3.8				
Secondary treatment performed (percent)^a^	11.4				
Secondary treatment (percent):^a^					
Open surgical/nephrectomy	33.3				
Endourological	25.0				
Conservative	0.0				
Interventional	41.7				
Concomitant injuries (percent):^a^					
Abdominal	22.6				
Rib fracture	21.7				
Other fracture	6.6				
Patients with high-grade renal trauma (percent)^a^	45.3				
Anticoagulation (percent)^a^	10.4				
Inpatient stay (days)	7.5	7	4.3	0	20
Intensive care (days)	1.9	1	3.9	0	35
Grade of renal trauma (*AAST*)	3.2	3	1.1	1	5
Number of packaged red blood cells in units	0.3	0	1.1	0	7
Total number of FU-CTs performed per patient	1.1	1	0.9	0	7
Days to first FU-CT	3.3	3	2.3	0	14
FU-CTs performed (percent)^a^	81.1				
FU-CTs relevant (percent)^a^	12.8				
Hb-level at admission	12.9	13.4	2.1	5.7	17.4
Hb-level at discharge	11.8	11.5	2.0	8.1	16.3
Difference in Hb-level	− 1.1	− 1.3	1.9	− 6	4.6
Cr-level at admission	1.1	1	0.3	0.6	3
Cr-level at discharge	1.0	0.9	0.3	0.5	2
Difference in Cr-level	− 0.1	− 0.1	0.3	− 1.7	0.7

*AAST* American Association for the Surgery of Trauma, *Cr* creatinine, *CT* computed tomography, *FU* follow-up, *Hb* hemoglobin, *SD* standard deviation

^a^Actual values were used during the estimations

Inclusion criteria were as follows: Blunt renal trauma, treated in the Clinic for Urology and Andrology between January 2010 and March 2020. Exclusion criteria were penetrating kidney trauma, < 15 and the treatment was conducted under the lead of another clinic. We included 106 patients with blunt renal trauma in this study. The mean age of patients was 43.0 years (female: 48 years, male: 42 years) and 83% were male (*n* = 88). In 65.1%, the renal trauma occurred on the left, and in 35.8% on the right side. One patient showed a double-sided renal trauma. The mean time of overall inpatient stay was 7.5 days and 1.9 days at the Intensive Care Unit (ICU).

The encountered renal traumata were classified according to the *American Association for the Surgery of Trauma* (*AAST*) as follows: 13 (12.3%) grade 1, 11 (10.4%) grade 2, 34 (32.1%) grade 3, 41 (38.7%) grade 4, and 7 (6.6%) grade 5. The median trauma grade was 3 [IQR = 1]. The renal trauma grades 4 and 5 were found in 45.3% of patients and were classified as “HG renal trauma”. Ten-point four percent of patients received anticoagulants. The following primary therapies were selected: conservative (56.6%), endourological (i.e., placement of Mono- or Double-J stent, 34.0%), operative (5.7%), and interventional (3.8%).

The reasons for endourological interventions were rupture of the ureter or renal pelvis, urinoma or hydronephrosis because of hematoma. The performed endourological interventions were placement of Mono-J stent and later change to Double-J Stent in 63.9% (*n* = 23) and immediate placement of Double-J stent in 36.1% (*n* = 13).

**Fig. 1:**
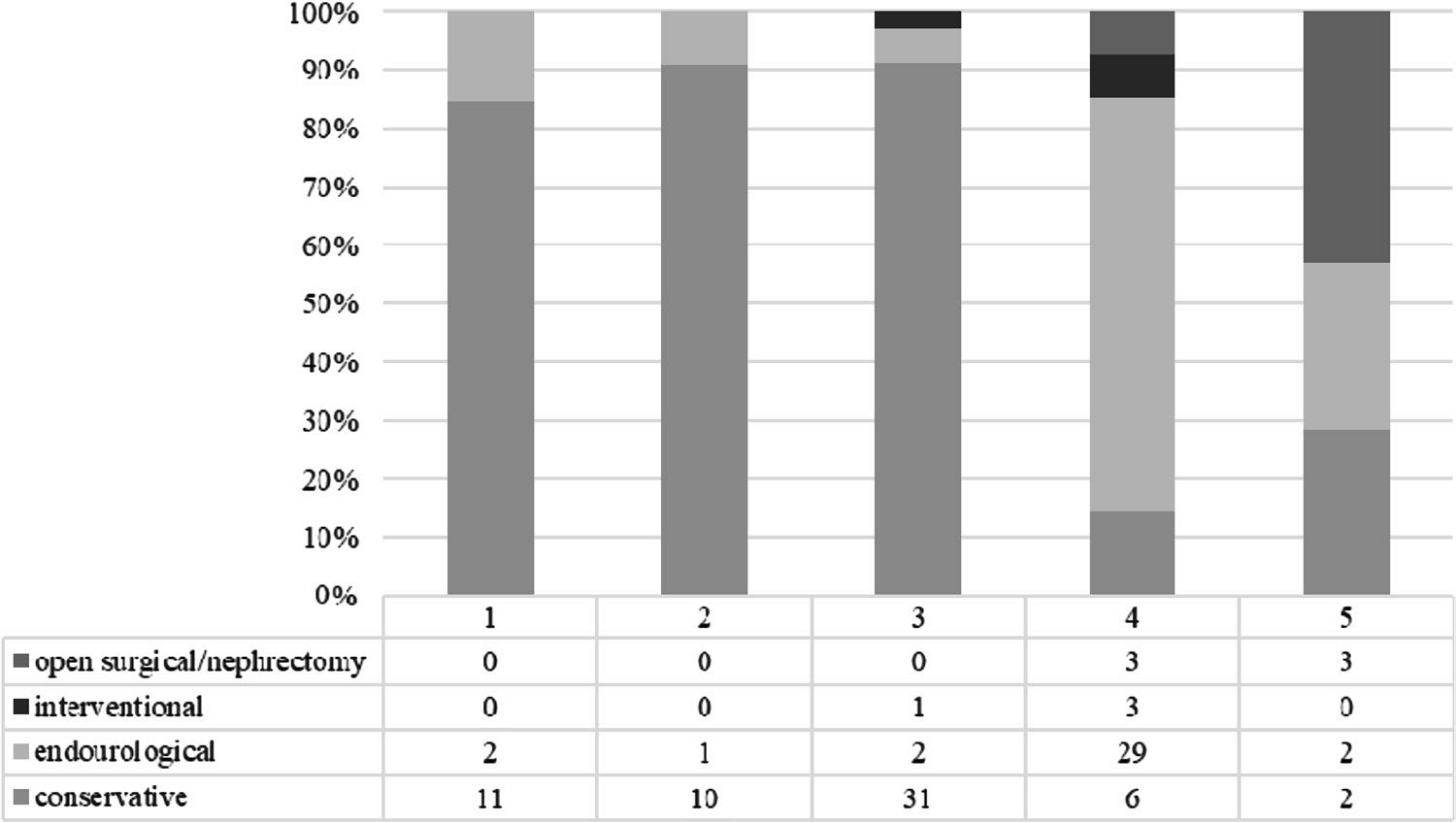
Primarily chosen therapies related to renal trauma grades 1–5 according to *American Association for the Surgery of Trauma (AAST)*

The selected primary therapies, subdivided according to trauma grade, can be found in Fig. 1.

Our standard treatment algorithm was as follows: all patients with suspected renal trauma received a CT scan with contrast agent. In the case of grade 1 and 2 blunt renal trauma, the stable patient was admitted to the normal ward. Patients with grade 3 or higher renal trauma were initially monitored in an ICU. In all patients with kidney trauma ≥ 3, the Hb value was determined at least once daily within the first 96 h after trauma.

In 81.1% of patients, an FU-CT was performed on average 3.3 days after the initial trauma. The average patient received 1.13 FU-CTs with a considerably high Standard Deviation (SD) of 1 and a maximum number of 7. It changed the primary therapy in 11.4%. Forty-one point seven percent of patients received an interventional, 25.0% an endourological (i.e., placement of Mono- or Double-J stent) and 33.3% an operative therapy (i.e., nephrectomy).

For Creatinine (Cr) levels, we found a mean of 1.1 mg/dl (96.8 umol/l) at admission and 1.0 mg/dl (88 umol/l) at discharge. Mean change in Cr-levels between admission and discharge was − 0.11 mg/dl (− 9.68 umol/l). The maximum increase in Cr between admission and discharge was 0.70 mg/dl (67.76 umol/l). None of the patients needed a temporary or permanent hemofiltration.

### Trauma mechanism

**Fig. 2:**
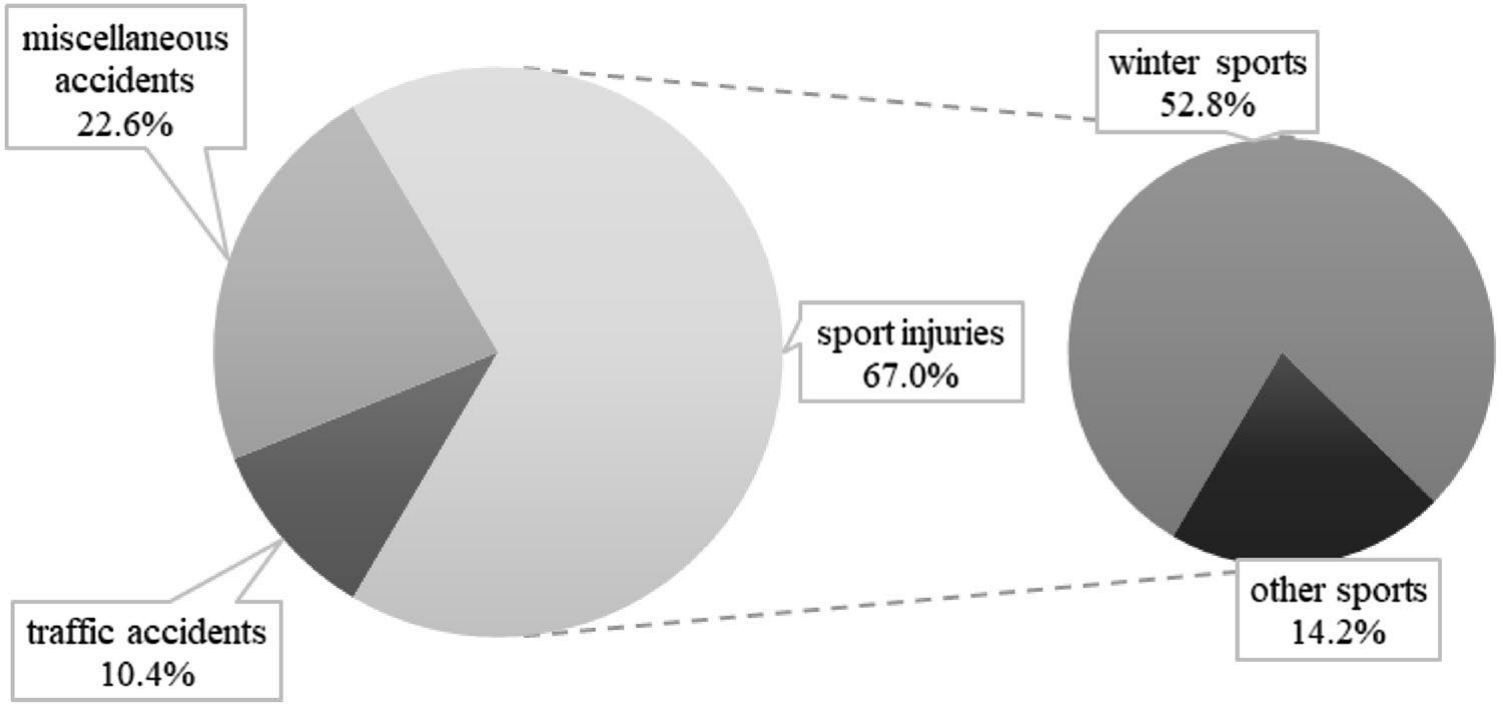
Mechanisms of injury (MOIs) of renal trauma in our patient collective (*N* = 106)

We subdivided the MOI as follows: winter sports, other sports (including racing and mountain bike and football accidents), traffic accidents (including motorbike and car accidents) and miscellaneous (including low falls and domestic animal injuries). Figure 2 summarizes the frequency of MOI in our collective. Among the *n* = 56 winter sports associated injuries, 71.4% (*n* = 40) were skiers, 26.8% (*n* = 15) were snowboarders, and 1.8% were sledders (*n* = 1), respectively.

### Methods

Logistic regression has been used to answer the following questions:

1. Is there a systematic sorting of patients who receive a FU-CT? Are certain patients more likely to receive a FU-CT based on their demographics or mechanism of injury?
2. What are significant predictors for a FU-CT influencing further treatment?
3. What are significant predictors for a nephrectomy?

A linear regression model was used to estimate the effects of various demographic and treatment-specific factors on a patient’s Hb-level at admission and discharge and its differences.

For the analysis of Hb loss, an unpaired *t* test with a confidence interval of 95% was performed.

Please refer to the supplementary material for explicit equations being used in this paper. Further information on the logit model and on odds ratios can be found in, e.g., [6].

## Results

### HG renal trauma and MOI

The frequency of HG renal trauma in sports associated injuries did not differ from the frequency in other MOIs (*p* = 0.84). Snowboarders, however, had more HG renal traumata compared to skiers (66.6% vs. 37.5%, *p* = 0.05).

### Concomitant injuries (CIs) and MOI

**Fig. 3:**
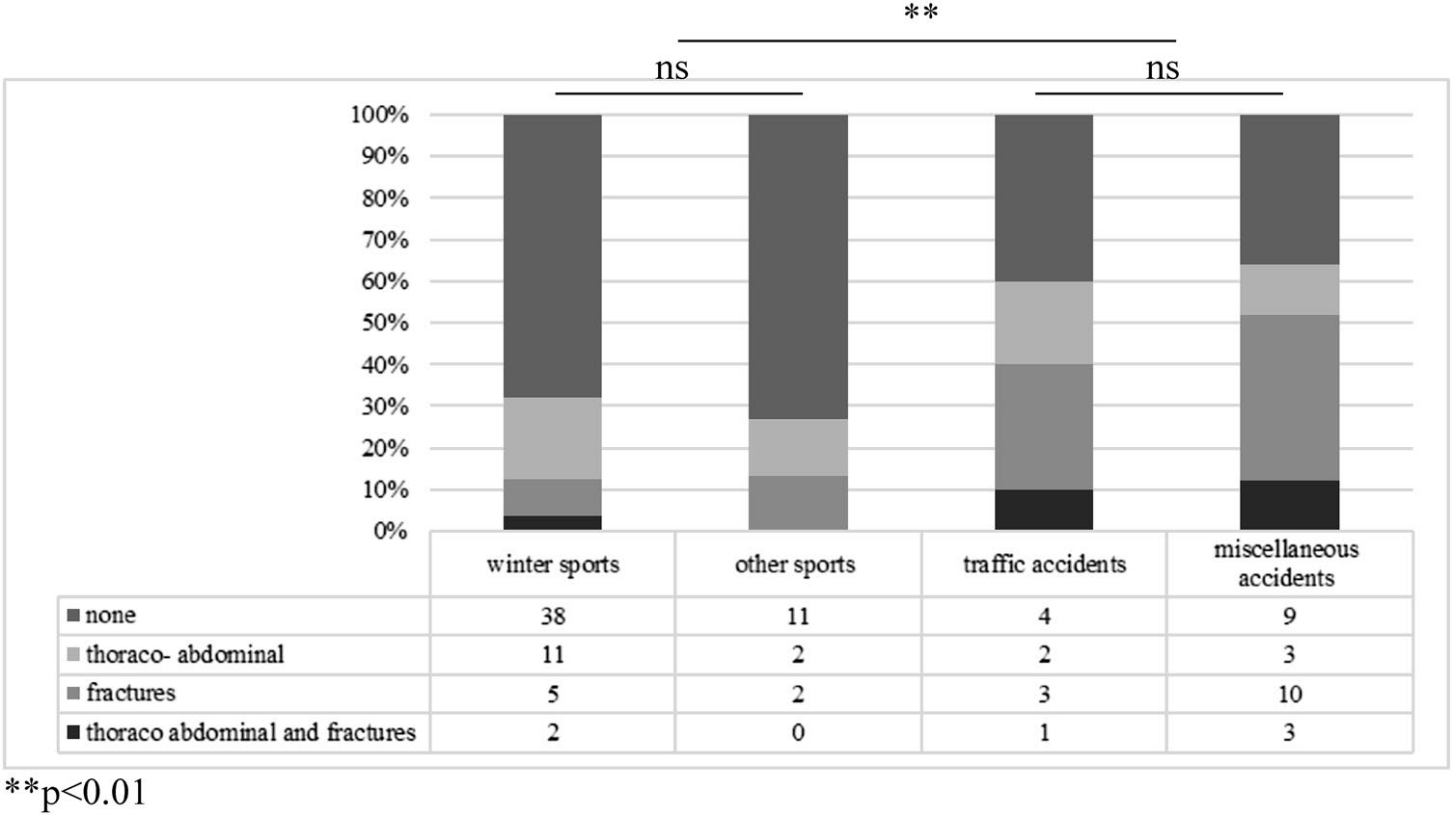
Concomitant injuries (CI) according to the mechanism of injury (winter sports *n* = 56, other sports *n* = 15, traffic accidents *n* = 10, miscellaneous accidents *n* = 25; statistical difference between the “CI: none” sections marked; ns = not significant)

Out of 106 patients, 62 (58.5%) had no CIs, 24 (22.6%) had thoraco-abdominal injuries (including spleen, liver, pancreas or lung lacerations), 23 (21.7%) had rib fractures, and 7 (6.6%) had other fractures. Looking at the MOIs independently, one finds differences between the groups in the frequency of CI. No CIs were detectable in 73.3%, 67.9%, 40.0%, and 36% in traumata associated to other sports, winter sports, traffic accidents and miscellaneous accidents, respectively. The CIs broken down according to the MOI can be found in Fig. 3. Comparing sports associated traumata with other MOIs, 69.0% (*n* = 49) vs. 37.1% (*n* = 13) had no CI (*p* < 0.01). In winter sports associated traumata, 73.3% of snowboarders and 65.0% of skiers had a solitary renal trauma (*p* = 0.75). However, a trend towards more frequent thoraco-abdominal CIs is found among snowboarders compared to skiers (36.4% vs. 26.9%, *p* < 0.5).

### Time of first FU-CT and rate of therapy change (ThC)

At least one FU-CT was performed in 81.1% of patients. The average time between diagnosis and the first FU-CT was 3.3 days. A mean number of 1.13 FU-CT scans was performed per patient. The longest period between initial trauma and first FU-CT was 14 days.

The results of a logistic regression analysis showed no significant relationship between the occurrence of HG renal trauma (*AAST* grade >  = 4) and the probability of receiving a FU-CT (OR 1.08, compare Table 1a in the supplementary material).

In 12 (11.4%) cases, the therapy changed after the FU-CT. Among these 12 cases were 2 (16.7%) grade 3, 9 (75.0%) grade 4 and 1 (8.3%) grade 5 renal trauma patients. There were 4 cases of secondary nephrectomy after various primary therapies, 1 grade 3 and 3 grade 4 traumata. Patients in need for a change in treatment were all symptomatic with an infection or drop of Hb-levels.

**Fig. 4:**
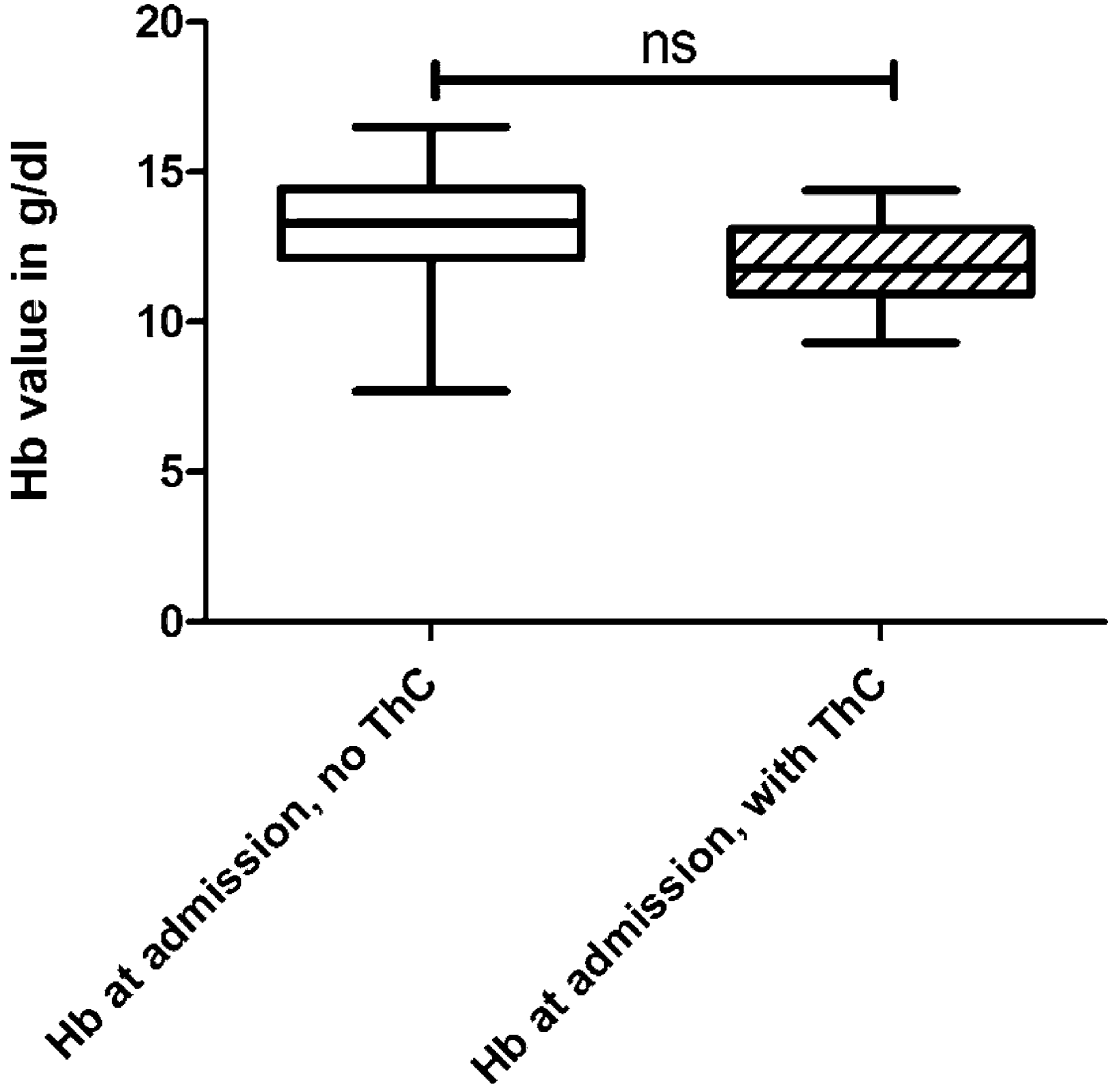
The initial hemoglobin (Hb) value at admission of all grade 3 and 4 kidney trauma patients with and without a therapy change (ThC). These values are not significantly different (*p* = 0.079)

**Fig. 5:**
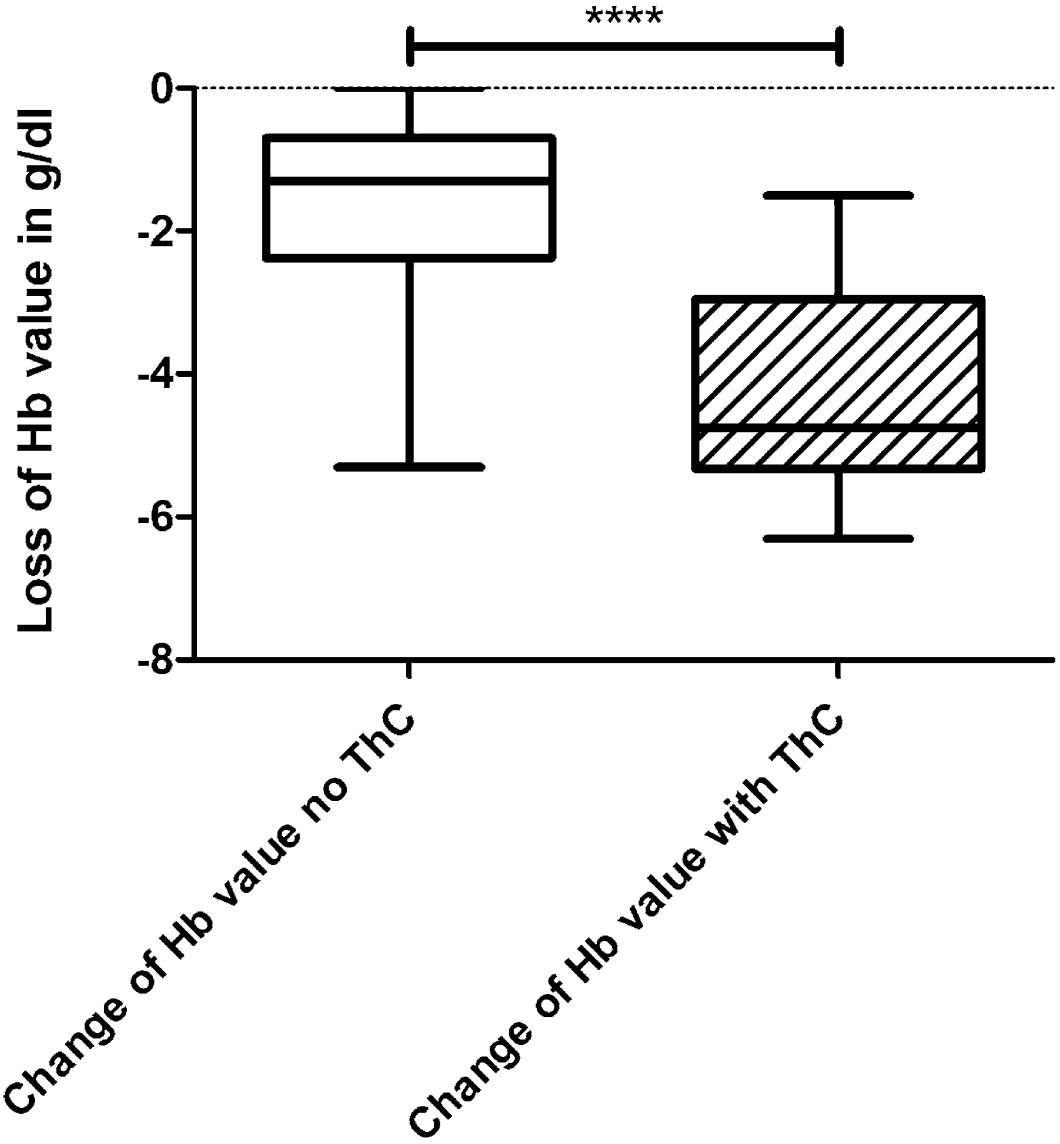
The drop of hemoglobin (Hb) value of the same groups as seen in Fig. 4 within 96 h and always before performing the ThC. This drop of Hb in the group with a ThC is significantly higher than in the control group (*p* < 0.0001)

We compared the groups with grade 3 and 4 renal traumata with and without revision surgery in terms of Hb loss within the first 96 h after trauma. Ten out of eleven patients with revision surgery were revised within this period. One developed an infection and we performed a nephrectomy 7 days after the initial trauma. This patient was not included in this statistical test. The analysis showed no differences between the groups of grade 3 and 4 trauma regarding the Hb value at admission. (*p* = 0.079). The further evaluation showed preoperatively a significantly increased Hb loss in the group of revised patients compared to the control group. In the grade 3 and 4 kidney trauma group without revision surgery (*n* = 64) the median Hb value at admission was 12.9 ± 0.23 g/dl with a median drop of Hb of 1.58 ± 0.16 g/dl. Compared to the group of grade 3 and 4 kidney trauma patients with change of treatment after FU-CT (*n* = 10) the median drop of Hb was 4.34 ± 0.46 g/dl, coming from 11.9 ± 0.89 g/dl, (*p* < 0.0001). The Hb values at admission and the change before a ChT of all grade 3 and 4 patients can be found in Figs. 4 and 5.

After nephrectomy (primary or secondary), no FU-CT was performed.

The question of interest is “In which case of renal trauma does a FU-CT make a difference in treatment?” Table 2a in the supplementary material summarizes the most important results for predictors of CT scan relevance. As the estimated intercept shows, the FU-CT had no relevance for the group of patients with renal traumata up to grade 3. For patients with a HG renal trauma, however, the FU-CT was on average significantly relevant for further treatment (*p* < 0.1). This means that only in case of patients with HG renal trauma, a FU-CT had a significant relevance in how the secondary treatment applied differed from the primary treatment. Neither the patient’s age (OR 0.99), nor the sex (OR 0.57), or the year of treatment (OR 1.29) were significant for a FU-CT’s relevance. However, for patients whose injuries resulted from winter sport accidents, a FU-CT was on average less relevant in terms of therapy changes (*p* < 0.05).

### Predictors of nephrectomy

Patients injured during winter sports or other sports were significantly less likely to receive a nephrectomy compared to other MOIs (min. *p* < 0.1) with no variation in factors. “HG trauma” was a powerful predictor of nephrectomy (*p* < 0.01). The same was true for the patient’s age (min. *p* < 0.05), the older the more likely. Furthermore, males were more likely to receive an open surgical treatment compared to women holding all other factors constant. Patients with fractures (excluding rib fractures), were less likely to receive an open, explorative surgery (*p* < 0.05). However, this was not true for all other CIs. The estimation results for a patient’s probability of receiving a nephrectomy can be found in the supplementary material, Table 3a.

### Inpatient stay and ICU

The MOI had no significant impact on the length of hospitalization (*p* = 0.61), the same was true for age (*p* = 0.94) and sex (*p* = 0.80). The length of inpatient stay significantly correlated with the presence of HG renal trauma (*p* < 0.001).

### Correlation between drop in hemoglobin (Hb) levels and renal trauma grade

The mean Hb level was 12.9 g/dl at admission and 11.8 g/dl at discharge for the reference group of female patients with renal trauma grade 1–3 who received no anticoagulants.

There was a strong correlation between the drop in Hb-level and the grade of renal trauma. Patients with HG renal trauma had a greater drop in Hb-levels during inpatient stay compared to low grade trauma patients (*p* < 0.05).

Neither age, nor that a patient took anticoagulants, had a significant effect on Hb-levels at both measurements. Hence, there is no significant driver to explain extraordinary drops of Hb-levels (please find the data in the supplementary material, Table 4a).

## Discussion

Our renal trauma collective has a unique characteristic compared to other collectives: the high percentage of sports, especially winter sports associated injuries. Amongst almost 16,000 US-American renal traumata (blunt and penetrating) analyzed by Hurtuk et al. [7] 73% of all cases were due to traffic accidents, 19% due to assaults and 8% because of miscellaneous causes (blows, firearm, suicides, machinery and piercing). In the over 10,000 blunt renal traumata cases analyzed by Voelzke et al. [4], the most common MOIs were traffic accidents (63%), followed by falls (14%) and sport activities (11%). In our study, sports with 67.3% and especially winter sports with 53.3% represents the most common MOI. Traffic accidents caused a renal trauma in only 10.3%.

Blunt renal traumata due to sports tend to occur isolated [8]: in a collective of 59 sports associated blunt renal trauma patients analyzed by Patel et al. [9], 69% had no CIs. In our study, the rate of patients with sports associated traumata without CIs was significantly higher compared to other MOIs (69.0% vs. 37.1%, *p* < 0.01). Analyzing the winter sports associated traumata individually, we found that in our collective the rate of patients without CIs in general was the same between the groups of skiers and snowboarders (*p* = 0.75). However, a trend towards an increased risk of additional abdominal injuries was evident for snowboarders, albeit with a small number of cases. Other authors also described an increased risk of abdominal injuries in snowboarders compared to skiers [10]. Geddes et al. [11] showed a six-fold increased risk of splenic trauma for snowboarders compared to skiers, with sharply rising risk in men. A look at the renal trauma grades also suggests that snowboarders suffer from more severe abdominal injuries: in our group of patients, snowboarders had a significantly higher risk of HG renal trauma compared to skiers.

Regarding the data of the FU-CT relevance in our collective, our results point in the same direction as the EAU guideline. It recommends FU-CT imaging 2–4 days after HG or penetrating renal trauma, or in case of symptoms like fever or flank pain [12]. Most often, trauma grades 4 and 5 are referred to as HG traumata. Hence, the course of disease varies and a clear definition will help to identify patients who benefit from FU-CTs [13].

According to Davis et al. [13] only 0.9% of patients without symptoms need a therapy change because of FU-CTs within the first 48 h. In contrast, 20.0% of patients with symptoms require an interventional treatment within the first 48 h after initial injury. Readmission rates are likewise increased in HG renal trauma patients [14].

The EAU guideline states that imaging can be safely omitted in asymptomatic grade 1–3 patients [12]. A congruent finding can be drawn from our data. In case of an asymptomatic clinical course in grade 1–3 blunt renal traumata, FU-CTs should be discussed critically as no therapeutic consequence resulted here in our collective. Some research groups even suggest to only perform symptom triggered re-imaging in cases of HG renal traumata: in a collective of 108 grade 4 blunt renal trauma patients analyzed by Loftus et al. [15], the rate of urologic complications in asymptomatic patients was not increased by omitting re-imaging. In addition, the data of Loftus et al. shows that 80% of patients who received an intervention following a routine FU-CT were asymptomatic. This leads to the assumption that FU-CTs may be unnecessary. The data of Hurtuk et al. [7] further strengthen this assumption. In his study, 91.5% of all nephrectomies after renal trauma took place within the first 12 h after the initial trauma. The question of the utility of the FU-CT gains even greater importance in regions where control imaging cannot be performed as regularly as in high-income economies. In a South African cohort of 74 renal traumata analyzed by Pillay et al. [16] only 28% of HG trauma patients received FU-CT. This was although none of the patients in this cohort had an isolated renal trauma, as most were polytraumatized. The authors note that 81% of HG renal trauma required nephrectomy. Thus, those patients who underwent a FU-CT were already among the most clinically stable, as the others had already undergone emergency laparotomies. Again, therapy is more likely to be triggered based on clinical signs, rather than on the findings of a control imaging. Predictors for nephrectomy in the South African collective were low Hb-levels, low systolic blood pressure, higher injury severity score, HG trauma, and age.

Looking at the interesting group of grade 3 and 4 kidney trauma patients, we found a significant correlation between the loss of Hb within a 96 h period after the trauma and the need of a change in treatment. The Hb loss was significantly higher in the group which received a revision therapy compared to the other group (*p* < 0.0001). This leads to our recommendation to measure the Hb value at least once a day for the first 96 h, regardless of the clinical condition of the patient. In this way, the danger of a continuing or newly occurring hemorrhage can be recognized at an early stage and further diagnostics can be carried out. For a clinically and Hb value stable renal trauma patient, the risk for nephrectomy is independent of its trauma grade and has virtually disappeared days after the initial trauma.

The median age of patients in our collective is 44 years and comparable to the literature [4, 7, 13, 17]. In such a young patient cohort, it would be preferable to avoid unnecessary radiation exposure: the typical radiation dose of an abdominal CT scan is 8 millisievert compared to 0.02 millisievert of a native chest radiography, or even to the average radiation exposure per person and year in Germany (2.1 millisievert).

There is a trend towards NOM in renal traumata: in an analysis of the American College of Surgeons’ National Trauma Data Bank performed by Hurtuk et al. [7], 93.8% of almost 16,000 renal traumata (blunt and penetrating) received a successful NOM. Data from Europe with over 8000 cases of renal traumata report a nephrectomy rate of 4% after blunt renal trauma [18]. In our study group 6 (5.7%) primary nephrectomies were performed. All others were treated by NOM (94.3%), including entirely conservative management, placement of ureteric stents and interventional techniques such as coil embolization. Four secondary nephrectomies because of complications (superinfection of renal hematoma/urinoma or drop of Hb-levels) were carried out. All were grade 3 or higher renal traumata. The overall nephrectomy rate in our study group was 9.4% and comparable to the collective of Hurtuk et al. [19]. Our findings are in line with the literature, suggesting NOM therapy standards even in grade 5 renal traumata, as long as patients are in a clinically stable condition. In our collective, the predictors of nephrectomy were “HG renal trauma”, age and sex. Other research groups showed a connection between grade 5 trauma and nephrectomy as well, whereas age was a predictor for complications after renal lacerations in the collective analyzed by McGuire et al. [20]. Another risk factor for surgical therapy described in the literature was hemodynamic instability, which was not evaluated in our collective. The requirement for blood transfusion, contrary to other studies, was not an independent risk factor in our study. In patients undergoing NOM, the overall survival and the rate of organ preservation is even better compared to patients undergoing surgical exploration, with no difference in complication rates between the two groups [21].

To safely perform NOM, close monitoring with regularly blood tests and serial re-evaluation for high-risk patients seems to be essential to detect clinical deterioration early on [22]. The equipment of a TC with easy access to diagnostics, blood banks, and specialized departments, such as interventional radiology, seems ideal for therapy planning. In our collective the mean ICU stay was 1.9 days, with the length of the total in-patient stay being significantly longer in kidney trauma grades 3 and upwards. The American Urological Association (AUA) suggests that NOM is a step-wise approach: Conservative treatment followed by endourological or interventional therapy, followed by surgical intervention if warranted [22].

The limitation of this study is its retrospective design and the lack of long-term follow-up data. Of special interest might be the evaluation of long term renal function outcomes, as data from patients post oncological nephrectomy shows that patients with concomitant diseases like diabetes or hypertension might suffer from a delayed renal function deterioration [23]. The development of hypertension seems to be a problem, especially in HG renal trauma patients.

## Conclusion

The data of our analysis showed that (winter) sports traumata are more likely to occur isolated and have a lower risk of severe outcomes, i.e., nephrectomy, compared to other MOIs. Snowboarders suffer from HG renal trauma and concomitant thoraco-abdominal injuries significantly more often than skiers.

FU-CT imaging in grade 1 and 2, as well as in asymptomatic grade 3 renal traumata, does not lead to a change in therapy and can, therefore, be safely omitted. However, logistic regression analysis showed that FU-CTs in grade 4 and 5 renal traumata are always appropriate, as the odds for a more radical treatment to follow are high.

In addition, the drop of Hb value should be checked daily for the first 96 h after trauma, as it was significantly higher in the group with grade 3 and 4 renal trauma with revision than in the group without.

Conclusively, we recommend in line with the EAU guideline [12] to perform a FU-CT on every symptomatic patient regardless of its renal trauma grade and on all trauma grades 4 and 5. A close monitoring is obligatory within the first 72–96 h after initial trauma. Across all trauma grades, implementing NOM is recommended.

## Supplementary Information

Below is the link to the electronic supplementary material.

Supplementary file1 (DOCX 37 KB)

## Data Availability

Data can be obtained from the authors on request.

## Abbreviations

AAST: American Association for the Surgery of Trauma
CI: Concomitant injury
Cr: Creatinine
CT: Computed tomography
EAU: European Association of Urology
FU: Follow-up
Hb: Hemoglobin
HG: High grade
ICU: Intensive care unit
MOI: Mechanism of injury
NOM: Non-operative management
SD: Standard deviation
TC: Trauma center
ThC: Therapy change

## Code availability

Code can be obtained from the authors on request.
