# Supplementary material for: On the black slope: analysis of the course of a blunt renal trauma collective in a winter sports region
Source: Eur J Trauma Emerg Surg. 2021 Dec 16;48(3):2125–33. doi: 10.1007/s00068-021-01830-w (PMC9192517; doi:10.1007/s00068-021-01830-w)
Supplement: Supplementary file 1 — Supplementary file1 (DOCX 37 KB) [file 68_2021_1830_MOESM1_ESM.docx]

**Supplementary Material: Statistical Methods**

Baseline equation of the logistic regression model

We estimated three adapted models using the following baseline equation of the logistic regression model:


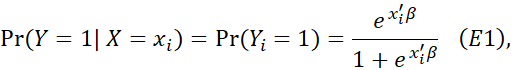


Where $Y_{i}$is a binary variable that takes the level 1 in case of an event and 0 otherwise for patient i? The event of interest could be, for example, a nephrectomy being performed. On the right-hand side, $x_{i}$ and denote a matrix of patient-specific characteristics, such as age or MOI and a vector of parameters containing the estimation results. In this study, we used odds ratios to interpret the estimated results meaningfully. Odds ratios can be directly derived from E1. To measure the probability $y_{i}=1$relative to the probability$y_{i}=0$, patient i’s relative risk for a specific event taking place, e.g. a nephrectomy, can be analyzed.


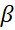

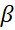


Predictors for a Follow Up (FU) Computed Tomography (CT) being relevant and predictors for nephrectomy

We tackle question 1-3 and re-formulate the baseline equation (E1) as follows:

$${Pr(cctp}_{i}=1)=\frac{e^{\beta_{0}+\beta_{1}hg_{i}+\gamma_{1}age_{i}+\gamma_{2}sex_{i}+\sum_{j=1}^{3} \delta_{j}tm_{i}+\sum_{l=1}^{3} \zeta_{l}ci_{i}+t}}{1+e^{\beta_{0}+\beta_{1}hg_{i}+\gamma_{1}age_{i}+\gamma_{2}sex_{i}+\sum_{j=1}^{3} \delta_{j}tm_{i}+\sum_{l=1}^{3} \zeta_{l}ci_{i}+t}} (E2)$$

$${Pr(cctr}_{i}=1)=\frac{e^{\beta_{0}+\beta_{1}hg_{i}+\gamma_{1}age_{i}+\gamma_{2}sex_{i}+\theta_{1}nct+\theta_{2}nct^{2}+\sum_{j=1}^{3} \delta_{j}tm_{i}+\sum_{l=1}^{3} \zeta_{l}ci_{i}+t}}{1+e^{\beta_{0}+\beta_{1}hg_{i}+\gamma_{1}age_{i}+\gamma_{2}sex_{i}+\theta_{1}nct+\theta_{2}nct^{2}+\sum_{j=1}^{3} \delta_{j}tm_{i}+\sum_{l=1}^{3} \zeta_{l}ci_{i}+t}} (E3)$$

$${Pr(neph}_{i}=1)=\frac{e^{\beta_{0}+\beta_{1}hg_{i}+\gamma_{1}age_{i}+\gamma_{2}sex_{i}+\sum_{j=1}^{3} \delta_{j}tm_{i}+\sum_{l=1}^{3} \zeta_{l}ci_{i}+t}}{1+e^{\beta_{0}+\beta_{1}hg_{i}+\gamma_{1}age_{i}+\gamma_{2}sex_{i}+\sum_{j=1}^{3} \delta_{j}tm_{i}+\sum_{l=1}^{3} \zeta_{l}ci_{i}+t}} \left( E4 \right)$$

On the left hand side of *E2-E4*, *cctp* and *cctr* stand for FU-CT performed and FU-CT relevant, respectively, while *neph* stands for nephrectomy performed. On the right hand side, $\beta_{0}$ denotes an intercept and $t$ is a time variable to control for potential time-dependent changes in treatment. We control for patients’ age and sex as well as for the trauma mechanism ($tm$) and concomitant injuries ($ci$). Furthermore, $hg$ adds a dummy containing information on the grade of renal trauma. If patient $i$ exhibits a renal trauma grade of 4 or 5 referring to *American Association for the Surgery of Trauma* (AAST), this is considered a high grade renal trauma and ${hg}_{i}= 1$ and 0 otherwise. Furthermore, we add the (squared) number of control CTs performed to E3 to control for an increasing probability of CT relevance in the number of CT images performed.

A linear regression model was used to estimate the effects of various demographic, trauma-and treatment-specific factors on a patient’s Hemoglobin (Hb)- levels at admission and discharge and their differences:

$${hb}_{i}=\beta_{0}+\beta_{1}hg_{i}+\gamma_{1}age_{i}+\gamma_{2}sex_{i}+\delta_{1}ac_{i} \left( E5 \right),$$

Hb denotes a patients Hb-level at admission, discharge or their difference. If patient $i$ received anticoagulation $ac_{i}=1$ and 0 otherwise. The remaining variables are the same used in *E2-E4*.

Test for sorting to rule out demographic or trauma factors as the main reason for a FU- CT (Table 1a)

We used equation E2 to estimate the predictors of a FU-CT being performed on patient i. Table 2 depicts the estimated results for variations of the model depicted in *E2*. We used four different specifications considering different variable sets of patient-specific characteristics. None of them indicated a significant influence of a patient’s demographic or trauma characteristics on the probability of receiving a FU-CT, except for concomitant rib fractures.

**Table 1a**: Test for sorting (CI= concomitant injury, MOI= mechanism of injury, SE= standard error).

| FU CT  (dummy) | (1) | | (2) | | (3) | | (4) |  |
| --- | --- | --- | --- | --- | --- | --- | --- | --- |
|  | Odds Ratios | SE | Odds Ratios | SE | Odds Ratios | SE | Odds Ratios | SE |
| (Intercept) | 4.48 | 0.98 | 4.34 | 1.00 | 9.81* | 1.28 | 8.12 | 1.40 |
| High-grade renal trauma (dummy) | 1.03 | 0.52 | 1.03 | 0.52 | 0.98 | 0.54 | 1.08 | 0.56 |
| Age | 1.00 | 0.01 | 1.00 | 0.01 | 0.99 | 0.01 | 1.01 | 0.02 |
| Sex | 0.85 | 0.70 | 0.87 | 0.71 | 1.00 | 0.74 | 1.32 | 0.81 |
| Year |  |  | 1.02 | 0.09 | 1.00 | 0.09 | 1.03 | 0.10 |
| MOI: winter sports |  |  |  |  | 0.52 | 0.78 | 0.35 | 0.88 |
| MOI: other sports |  |  |  |  | 0.86 | 1.00 | 0.62 | 1.08 |
| MOI: traffic accidents |  |  |  |  | 0.22 | 0.96 | 0.27 | 1.03 |
| CI: abdominal |  |  |  |  |  |  | 0.99 | 0.64 |
| CI: rib fracture |  |  |  |  |  |  | 0.20 ^**^ | 0.70 |
| CI: other fracture |  |  |  |  |  |  | 2.55 | 1.28 |
| Observations | 106 | | 106 | | 106 | | 106 |  |
| R^2^ Tjur | 0.001 | | 0.001 | | 0.032 | | 0.093 |  |
| * p<0.1   ** p<0.05   *** p<0.01 | | | | | | |  |  |

**Table 2a:** Test for follow- Up computed tomography (FU-CT) relevance (CI= concomitant injury, MOI=mechanism of injury, SE= standard error).

| Control CT relevant (dummy) | (1) | | (2) | | (3) | | (4) | |
| --- | --- | --- | --- | --- | --- | --- | --- | --- |
|  | Odds  Ratios | SE | Odds  Ratios | SE | Odds  Ratios | SE | Odds  Ratios | SE |
| (Intercept) | 0.06 ** | 1.45 | 0.00 *** | 3.55 | 0.00 *** | 4.45 | 0.00 ** | 4.58 |
| High-grade renal trauma (dummy) | 4.96 * | 0.88 | 9.15 * | 1.13 | 15.49 * | 1.43 | 11.41 * | 1.40 |
| Year | 1.07 | 0.13 | 1.11 | 0.15 | 1.26 | 0.20 | 1.29 | 0.21 |
| Age | 1.00 | 0.02 | 1.02 | 0.02 | 0.98 | 0.03 | 0.99 | 0.04 |
| Sex | 0.27 * | 0.79 | 0.31 | 0.95 | 0.39 | 1.23 | 0.57 | 1.32 |
| Number of FU-CTs performed | 1.91 * | 0.38 | 610.26 ** | 2.53 | 78392.90 *** | 4.19 | 55363.98 ** | 4.42 |
| Number of FU-CTs performed^2^ |  |  | 0.35 * | 0.56 | 0.12 ** | 0.90 | 0.14 ** | 0.98 |
| MOI: winter sports |  |  |  |  | 0.01 ^**^ | 2.10 | 0.01 ^**^ | 2.13 |
| MOI: other sports |  |  |  |  | 0.13 | 1.97 | 0.11 | 2.11 |
| MOI: traffic accidents |  |  |  |  | 0.11 | 2.59 | 0.12 | 2.95 |
| CI: abdominal |  |  |  |  |  |  | 0.76 | 1.25 |
| CI: rib fracture |  |  |  |  |  |  | 0.19 | 2.51 |
| CI: other fracture |  |  |  |  |  |  | 3.30 | 2.08 |
| Observations | 86 | | 86 | | 86 | | 86 | |
| R^2^ Tjur | 0.347 | | 0.397 | | 0.575 | | 0.584 | |
| * p<0.1   ** p<0.05   *** p<0.01 | | | | | | | | |

Table 3a: Predictors of nephrectomy (MOI= mechanism of injury, CI= concomitant injury)

| **Nephrectomy performed (dummy)** | **(1)** | | **(2)** | | **(3)** | | **(4)** | |
| --- | --- | --- | --- | --- | --- | --- | --- | --- |
|  | Odds Ratios | SE | Odds  Ratios | SE | Odds  Ratios | SE | Odds  Ratios | SE |
| (Intercept) | 0.09 *** | 0.47 | 0.00 *** | 1.71 | 0.00 *** | 1.83 | 0.00 *** | 2.39 |
| High-grade renal trauma (dummy) | 32.25 *** | 0.58 | 195.31 *** | 0.99 | 346.11 *** | 1.17 | 947.68 *** | 1.46 |
| Year | 1.03 | 0.09 | 1.09 | 0.10 | 1.14 | 0.12 | 1.10 | 0.12 |
| Age |  |  | 1.06 *** | 0.02 | 1.05 ** | 0.02 | 1.07 ** | 0.03 |
| Sex |  |  | 3.61 | 0.82 | 5.67 ** | 0.85 | 8.08 ** | 0.98 |
| MOI: winter sports |  |  |  |  | 0.20 | 0.99 | 0.14 * | 1.07 |
| MOI: other sports |  |  |  |  | 0.09 * | 1.26 | 0.05 ** | 1.39 |
| MOI: traffic accidents |  |  |  |  | 0.12 | 1.34 | 0.10 | 1.47 |
| CI: abdominal |  |  |  |  |  |  | 1.48 | 0.85 |
| CI: rib fracture |  |  |  |  |  |  | 2.10 | 1.02 |
| CI: other fracture |  |  |  |  |  |  | 0.03 ** | 1.46 |
| Observations | 106 | | 106 | | 106 | | 106 | |
| R2 Tjur | 0.487 | | 0.563 | | 0.580 | | 0.619 | |
| * p<0.1   ** p<0.05   *** p<0.01 | | | | | | | | |

Table 4a: impact of various parameters on Hemoglobin (Hb)- levels (SE= standard error).

| **Hb-level** | **Admission** | | **Discharge** | | **Loss** | |
| --- | --- | --- | --- | --- | --- | --- |
|  | Estimates | SE | Estimates | SE | Estimates | SE |
| (Intercept) | 12.46 ^***^ | 0.78 | 11.68 ^***^ | 0.73 | -0.78 | 0.72 |
| High-grade renal trauma (dummy) | -0.59 | 0.42 | -0.84 ** | 0.39 | -0.25 | 0.39 |
| Age | -0.01 | 0.01 | -0.01 | 0.01 | 0.01 | 0.01 |
| Sex | 1.60 ^***^ | 0.53 | 0.94 * | 0.50 | -0.66 | 0.49 |
| Anticoagulation | -0.41 | 0.71 | 0.00 | 0.67 | 0.41 | 0.66 |
| Observations | 104 | | 104 | | 104 | |
| R^2^ / R^2^ adjusted | 0.134 / 0.099 | | 0.086 / 0.049 | | 0.044 / 0.005 | |
| * p<0.1   ** p<0.05   *** p<0.01 | | | | | | |
